# Upcycled Beverage From Roasted Açaí (*Euterpe oleracea*) Seeds: Antioxidant Capacity and Cytoprotection Through Gastrointestinal Simulation

**DOI:** 10.1002/mnfr.70270

**Published:** 2025-09-16

**Authors:** Layane Rosa da Silva, Luis Henrique Felix Feitosa, Matheus Bezerra e Silva, Gezaildo dos Santos Silva, Eike Guilherme Torres de Souza, Antonia Dayane Jenyffer de Farias Marques, Eduarda Spagnol Sacilotto, Narciza Maria de Oliveira Arcanjo, Marcos dos Santos Lima, Maria Teresa Bertoldo Pacheco, Fabiana Galland, Marta Suely Madruga, Ana Rita Ribeiro de Araújo Cordeiro, Taliana Kênia Alencar Bezerra

**Affiliations:** ^1^ Department of Food Engineering, Technology Centre Federal University of Paraíba João Pessoa Brazil; ^2^ Food Technology Institute (ITAL). Science and Food Quality Center, São Paulo Campinas Brazil; ^3^ Department of Food Technology Federal Institute of Sertão Pernambucano Petrolina Campus, Beverage Technology Laboratory, Jardim São Paulo Petrolina Brazil; ^4^ Food Department Technology, Federal Institute of Amapá COTGALI, IFAP Macapá Brazil

**Keywords:** açaí seeds, bioactive compounds, cytoprotective effect, oxidative stress, simulated digestion

## Abstract

Acai seeds have been used in roasting processes, resulting in a percolated beverage similar to coffee. This upcycled product has gained market share, offering consumers a caffeine‐free beverage with functional appeal. However, information about the powder and beverage is limited. Thus, this research aimed to evaluate the chemical profile of roasted acai seed samples from Brazil, investigate phenolic compounds and antioxidant activity before and after in vitro digestion, and examine the cytotoxic/cytoprotective effects in Caco‐2 cell cultures. Six commercial samples from the North, Northeast, and Southeast were characterized by high carbohydrate content, high acidity, and moisture content according to legislation (≤5 g/100 g). After in vitro digestion, all beverages exhibited higher concentrations of tannins and flavonoids and greater capacity to absorb oxygen radicals. However, there was a reduction in antioxidant potential against DPPH and ABTS radicals and in the reducing action of ferric ions. The samples produced in the North and Northeast showed similarity in the phenolic profile compared to those made in the Southeast. They were characterized by chlorogenic acids, vinylic acid, and syringic acid, which are associated with phenolic markers. All beverages demonstrated low cytotoxicity (>90%) and showed cytoprotective effects, indicating that the samples prevented oxidation.

## Graphical abstract

Roasted açaí grains contain phenolic compounds, including chlorogenic and vinylic acids, catechin, tannins, and procyanidins. Their percolates show antioxidant potential after in vitro digestion, with low cytotoxicity and cytoprotective effects on Caco‐2 cells, highlighting their bioactive value.

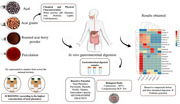

**Abbreviations**
AAPH: 2,2'‐Azobis(2‐amidinopropane)dihydride
ABTS: 2,2‐azino‐bis‐3‐ethylbenzothiazoline‐6‐sulfonic acid
AlCl3: aluminum chloride
ANVISA: National health surveillance agency
CaCl_2_
: calcium chloride
DCF‐DA: dichlorodihydrofluorescein diacetate
DMSO: dimethyl sulfoxide
DPPH: 3 2,2‐diphenyl‐1‐picrylhydrazyl
FeCl_3_
: ferric chloride
FRAP: iron‐reducing antioxidant power
GAE/g: grams of gallic acid equivalents
H_2_O_2_
: hydrogen peroxide
HCA: hierarchical clustering analysis
HCl: hydrochloric acid
MTT: (3‐(4,5‐Dimethylthiazol‐2‐yl)‐2,5‐iphenyltetrazolium Bromide)
N: north
Na_2_CO_3_
: sodium carbonate
NaNO_2_
: sodium nitrite
NaOH: sodium hydroxide
N‐AP: sample produced in the North‐ Amapá
NE: northeast
NE‐PE: sample produced in the Northeast—Pernambuco
ORAC: oxygen radical absorbance capacity
PC: principal component analysis
pH: potential of hydrogen
ROO: reduce peroxyl radicals
ROS: reactive oxygen species
SE: southeast
SE‐SP: sample produced in the Southeast—São Paulo
TAE/g: antioxidant activity of grams
TE/g: Trolox equivalent
TEAC: Trolox equivalent antioxidant capacity
TPTZ: 2,4,6‐Tris(2‐pyridyl)‐s‐triazine

## 1 Introduction

The growing demand for sustainable food production models has led to the development of upcycled foods, which add value to industrial by‐products, apply circular economy principles, and reduce food waste [1]. Among these, açaí (*Euterpe oleracea* Mart.) stands out, a palm tree native to the Amazon rainforest, with Brazil being the leading producer and consumer in the world, and the State of Pará responsible for approximately 94% of this production [2].

Açaí pulp has been widely consumed due to its high nutritional value and relevant sensory attributes, such as flavor. It is also notable for being a source of bioactive compounds with high antioxidant capacity due to phenolic compounds such as flavonoids, tannins, anthocyanins, and fatty acids [3]. However, this fraction represents only 5% to 15% of the total fruit, and the remaining portion consists of by‐products resulting from processing, such as fibers and seeds, which, although underutilized, show increasing evidence of their functional potential [4].

Açaí seeds (in natura) exhibit a rich chemical and bioactive composition, associated with cardiovascular, renal, and metabolic protection, as well as with reduced risks of obesity, hyperglycemia, and oxidative stress (Mendonça et al., 2024). Studies have highlighted the bioactive potential of açaí seeds due to the action of phenolic compounds, particularly gallic acid, catechin, epicatechin, tannins, and soluble fibers such as inulin [4, 5].

An emerging application refers to the use of açaí seeds açaí in roasting processes, which result in the production of a powder to be used in the preparation of percolated beverage, similar to coffee. This upcycled product has gained market share due to the recognized benefits of açaí, offering consumers a caffeine‐free beverage with functional appeal [5]. However, the lack of standardization regarding the parameters of the roasting process, especially time and temperature, combined with the artisanal way of doing it, impacts the final product's chemical composition and sensory and bioactive characteristics [6].

Scientific research that chemically characterizes the beverages obtained from roasting açaí seeds and their quality and bioactive potential is still scarce. Thus, studies on the functional properties of this powder and the beverage obtained, which elucidate its biological potential and evaluate the stability and bioaccessibility of the phenolic compounds after simulated digestion, are essential to substantiate the functional claims and establish it as a new food product.

In this sense, the cytotoxic and cytoprotective effects on intestinal cells are still being investigated. They were not explored. Tests with intestinal cell models, such as Caco‐2 cells, are essential to evaluate how the intestinal epithelium absorbs nutrients and interacts with bioactive compounds [7].

Therefore, the objective of the study was to evaluate beverages made with roasted açaí beans, determining physicochemical parameters, bioactive evaluation before and after digestion (in vitro), antioxidant activity, and cytotoxic/cytoprotective effects in Caco‐2 cell cultures, produced and marketed in different regions of Brazil (North, Northeast, and Southeast).

## 2 Experimental Section

### 2.1 Sample Collection and Physical‐Chemical Characterization

Six samples of roasted açaí grain powder from the local market, produced in three Brazilian regions, were collected: North (four), Northeast (one), and Southeast (one). The number of samples per region reflected product availability and was intentional to represent variability in processing practices. After collection, the roasted açaí bean powders were evaluated for physical and chemical parameters (pH, water activity, moisture, proteins, lipids, ashes, and carbohydrates) using [8] methodologies, except carbohydrates, calculated by difference.

### 2.2 Elaboration of Percolated Beverage of Roasted Açaí Grains

Percolated beverages were prepared from each roasted açaí seed powder using a 1:10 (w/v) ratio of powder and hot water at 100°C, extracted by the percolation method with a paper filter. The beverages were collected, transferred to sterile Falcon tubes, and stored under refrigeration at 4°C ± 1°C until subsequent analyses. The extraction yield, calculated as the percentage of beverage obtained relative to the initial mass of roasted açaí seed powder and water volume used, was 90%.

### 2.3 Screening of Samples

One sample per region was selected based on total phenolic content (Table S1) determined by the Folin–Ciocalteu method proposed by Andrade et al. [9] to maintain regional representation. The samples from the North region showed phenolic content of 1.68 to 2.60 mg GAE/g of roasted açaí bean powder, while the sample produced in the Northeast region presented 1.72 mg GAE/g of powder, and the sample from the Southeast region presented 1.97 mg GAE/g of powder. The three samples with the highest total phenolic content were statistically selected to continue the bioactive study, with N‐AP representing the North, NE‐PE Northeast, and SE‐SP Southeast.

### 2.4 In Vitro Simulated Digestion

Simulated gastrointestinal digestion was performed as described by the Infogest group [10] in three phases (oral, gastric, and intestinal) with specific enzymes and fluids. The pH of the fluids was adjusted using 1 M HCl or 1 M NaOH. Two mL of each sample were placed in 50 mL Falcon tubes and incubated at 37°C in a stirred incubator (SP‐222, SPLABOR, São Paulo, Brazil). Oral digestion was performed by mixing the samples with 1.6 mL of salivary fluid, 10 µL of 0.3 M CaCl_2_, 0.05 mL of 75 U/mL α‐amylase, and 0.39 mL Milli‐Q water to reach 4 mL, incubating for 2 min. The pH was then adjusted to 3.0, and the volume was completed with Milli‐Q water. Samples were incubated for 2 h. For intestinal digestion, intestinal fluid containing 5 mL pancreatin solution (100 U/mL), 2.5 mL 10 mM biliary solution, and 16 µL of 0.3 M CaCl_2_ was used, followed by 2 h incubation. A sample‐free control was performed under the same conditions. At each digestion stage, 2 mL of each sample and control were collected, centrifuged at 3500 × *g* for 15 min, and the supernatants were stored at −20°C ± 2°C for analysis.

## 3 Bioactive Evaluation Before and After Digestion (in vitro)

### 3.1 Total Flavonoids

The determination of total flavonoids was performed using the methodology proposed by Zhishen et al. [11]. A 100 µL aliquot of the percolate was homogenized with 150 µL of 5% (m:v) NaNO_2_ and left to react for 5 min. Then, 150 µL of 10% (m:v) AlCl_3_ was added and left to react for 6 min. Finally, 1000 µL of 1 M NaOH and 1200 µL of distilled water were added. The reading was performed by spectrophotometry (Quimis, Q798U, São Paulo, Brazil) at 510 nm. The result was expressed in mg of catechin (EC) per g of sample.

### 3.2 Condensed Tannins

Tannin determination was performed using the Folin–Ciocalteu method. Aliquots of 100 µL of percolation, 1000 µL of Na_2_CO_3_ solution, 8400 µL of distilled water, and 500 µL of Folin radical were pipetted, completing 10 mL of final volume. After homogenization, the samples were kept at 25°C for 30 min, and then readings were taken on a UV/Visible spectrophotometer (Quimis, Q798U, São Paulo, Brazil) at 725 nm. The result was expressed in mg of tannic acid (TAE) per g of sample [12].

### 3.3 Diphenyl‐1‐Picrylhydrazyl (DPPH) Radical Scavenging Activity

The DPPH⁺ radical scavenging capacity of roasted açaí grain percolates was determined according to Brand‐Williams et al. [13]. A 100 µL extract aliquot was added to 3.9 mL DPPH⁺ in methanol. After homogenization, the blank containing free radical and solvent was read at time zero, and tests were maintained at 25°C for 30 min. The scavenging activity was measured at 515 nm in a UV‐VIS spectrophotometer (Quimis, Q798U, São Paulo, Brazil), and capacity was calculated. The calibration curve was prepared with Trolox (50–1000 µM), and results were expressed as µmol Trolox equivalent (TE) per gram of sample (µmol TE/g).

### 3.4 Trolox Equivalent Antioxidant Capacity (TEAC)

The ABTS⁺ radical scavenging capacity was determined according to Re et al. [14]. A 30 µL sample aliquot was added to 3000 µL ABTS⁺ in methanol. After homogenization, the blank containing free radical and solvent was read at time zero, and triplicate tests were maintained at 25°C for 6 min. Activity was measured at 734 nm in a UV‐VIS spectrophotometer (Quimis, Q798U, São Paulo, Brazil), zeroed with ethanol. The calibration curve was prepared with Trolox (100–2000 µM), and results were expressed as µmol Trolox equivalent (TE) per gram of sample (µmol TE/g).

### 3.5 Iron‐Reducing Antioxidant Power (FRAP)

The ability to reduce iron ions was evaluated using [15] methodology. The FRAP reagent was prepared by mixing acetate buffer (300 mmol/L, pH 3.6), 10 mmol/L TPTZ in 40 mmol/L HCl, and 20 mmol/L FeCl_3_ at 10:1:1 (v:v:v). A 90 µL extract aliquot was added to 270 µL distilled water and 2.7 mL FRAP reagent, homogenized, and incubated at 37°C for 30 min. The reduction of Fe^3^⁺ to Fe^2^⁺ was measured at 595 nm in a UV‐VIS spectrophotometer (Quimis, Q798U, São Paulo, Brazil), using solvent with FRAP reagent as blank. Results were expressed as mg Trolox equivalent (TE) per gram (mg TE/g), based on a Trolox calibration curve (50–1000 µM).

### 3.6 Oxygen Radical Absorbance Capacity—ORAC

The ORAC assay evaluates antioxidant ability to reduce peroxyl radicals (ROO^−^) generated by thermal degradation of AAPH and protect fluorescein from oxidation [16]. Samples and Trolox standards (5–80 µM) were diluted in 75 mM potassium phosphate buffer (pH 7.4). In a microplate, 20 µL sample or standard, 120 µL 0.17 µM fluorescein, and 60 µL 40 mM AAPH were added. Fluorescence was monitored for 2 h at 90‐s intervals (excitation 485 nm, emission 520 nm) using a microplate reader (Varioskan Lux, Thermofisher, Singapore). Sample and standard protection (AUCnet) were calculated as the difference between area under fluorescence decay curves of samples/standards and blanks. Results were expressed as µmol Trolox equivalent/g sample and as percentage variation between ORAC values.

## 4 Phenolic Profile

### 4.1 Determination of Phenolic Profile

The phenolic compound profile was determined following Padilha et al. [17] using an Agilent 1260 Infinity LC System coupled to a Diode Array Detector (model G1315D). Data were processed with OpenLAB CDS ChemStation software. The column was Zorbax Eclipse Plus RP‐C18 (100 × 4.6 mm, 3.5 µm) with a Zorbax C18 pre‐column (12.6 × 4.6 mm, 5 µm). Injection volume was 20 µL, and solvent flow was 0.8 mL/min. The gradient was 0–5 min: 5% B; 5–14 min: 23% B; 14–30 min: 50% B; 30–33 min: 80% B; solvent A was phosphoric acid solution (pH 2.0), and solvent B was methanol acidified with 0.52% phosphoric acid. Samples were prepared and filtered through a PVDF membrane filter (13 mm diameter, 0.45 µm pore size).

Compounds were detected at 220, 280, 320, 360, and 520 nm, and identification was performed by comparing the retention times of the sample peaks with those of the external standards and comparing the UV spectral similarity between the peaks and the external standards. Quantification was performed using calibration curves. The method validation parameters are presented in Table S2.

### 4.2 Cytotoxic/Cytoprotective Assays (Caco‐2 Cell Culture)

#### 4.2.1 Culture and Maintenance Conditions of Caco‐2 Cells

The Caco‐2 cell line, a human adenocarcinoma cell line derived from human intestinal epithelium, was acquired from the Cell Bank of Rio de Janeiro (code 0059). Cells were cultured in 25 cm^3^ flasks with DMEM medium supplemented with 10% fetal bovine serum, 1% penicillin/streptomycin, 8.4 mM HEPES, 1% sodium pyruvate, 1% non‐essential amino acids, and 1% L‐glutamine. The cultures were maintained at 37°C under a 5% CO_2_ atmosphere, and the medium was refreshed every 2 days.

#### 4.2.2 Evaluation of Cell Viability With MTT Reduction Assay

For experiments, exponentially growing Caco‐2 cells were trypsinized and seeded into 96‐well plates at a density of 2 × 10⁴ cells/well. Once confluent, the cells were pre‐treated for 1 h with extracts (N, NE, SE) at a concentration of 5 ng/mL. This was immediately followed by a 1‐h treatment with either the samples alone or the samples in the presence of 1 mM hydrogen peroxide (H_2_O_2_).

Cell viability assay was performed using the MTT ((3‐(4,5‐Dimethylthiazol‐2‐yl)‐2,5‐iphenyltetrazolium Bromide) assay. Cells were incubated with 50 µg/mL MTT for 30 min at 37°C. Then, the medium was removed, and the purple MTT crystals were dissolved in DMSO under shaking. Absorbance values were measured at 560 and 650 nm (Varioskan Lux, Thermofisher, Singapore) and expressed as a percentage of basal.

#### 4.2.3 Evaluation of ROS Production With DCF‐DA Assay

Reactive oxygen species (ROS) production was evaluated by the DCF‐DA assay after cell treatment [18]. DCF‐DA (20 µM) was incubated for 30 min before the treatment ended with samples at different concentrations (mg/mL). Cells were washed twice with PBS, then 100 µL of 1 mM hydrogen peroxide or basal medium was added. The microplate was placed in a microplate reader (Varioskan Lux, Thermofisher, Singapore) for fluorescence reading (excitation 485 nm, emission 520 nm) every 5 min for 2 h at 37°C. Results were expressed as arbitrary fluorescence units per microgram of protein (UF/mg).

#### 4.2.4 Statistical Analysis

All analyses were performed in triplicate. The results obtained were subjected to analysis of variance (ANOVA) and compared using the Tukey test or *t*‐test with a probability of error of 5%. Hierarchical cluster analysis (HCA) was performed using a graphical interface [19].

## 5 Results and Discussion

### 5.1 Physicochemical Parameters of Roasted Açaí Grain Powders

The roasted açaí bean powders showed substantial variability in their chemical composition (Table 1), which is probably due to the lack of standardization of the roasting process within the Brazilian regions, climatic factors, and a variety of raw materials.

**TABLE 1:** Physico‐chemical characterizations of roasted açaí grains from different regions of Brazil.

Samples	Parameters						
	Moisture	Ash	Proteins	Lipids	Carbohydrates	Aw	pH
NE(PE)	4.71 ± 0.16^b^	1.28 ± 0.02^d^	5.38 ± 0.14^ab^	5.09 ± 0.42^d^	89.76 ± 0.37^a^	0.41 ± 0.006^c^	4.36 ± 0.28^b^
N(AP1)	2.18 ± 0.11^e^	0.83 ± 0.07^e^	5.06 ± 0.01^bc^	11.41 ± 0.99^a^	83.53 ± 0.98^cd^	0.28 ± 0.006^e^	4.18 ± 0.16^b^
N(AP2)	3.06 ± 0.17^d^	1.45 ± 0.07^c^	5.06 ± 0.30^bc^	11.08 ± 1.00^b^	83.86 ± 1.08^cd^	0.38 ± 0.004^d^	4.16 ± 0.04^b^
N(AP3)	5.30 ± 0.02^a^	0.96 ± 0.016^e^	5.53 ± 0.31^ab^	12.22 ± 0.13^a^	82.25 ± 0.23^d^	0.45 ± 0.0008^b^	4.14 ± 0.12^b^
N(PA)	3.60 ± 0.14^c^	1.92 ± 0.10^a^	5.67 ± 0.03^a^	9.19 ± 0.24^c^	85.14 ± 0.21^c^	0.55 ± 0.002^a^	4.94 ± 0.13^a^
SE(SP)	3.87 ± 0.11^c^	1.63 ± 0.07^b^	5.42 ± 0.30^b^	12.39 ± 0.05^a^	82.19 ± 0.17^d^	0.41 ± 0.002^c^	2.95 ± 0.11^c^

*Note*: NE(PE): Northeast (Pernambuco). N(AP1): North (Amapá 1). N(AP2): North (Amapá 2). N(AP3): North (Amapá 3). N(PA): North (Pará). SE(SP): Southeast (São Paulo). The results were expressed in (g/100 g) of sample with mean ± standard deviation. Different superscript lowercase letters in the same column, for the same method, denote differences (*p* ≤ 0.05), based on Tukey's test.

The moisture content ranged from 1.52 to 5.30 g/100 g, within the general limit for roasted açaí beans (≤5%) [20]. The moisture levels are a consequence of the time and rigor of roasting the beans, which results in weight loss, being responsible for the higher or lower water content in the final product [21]. Similar values ​​were observed for the water activity content, which ranged from 0.14 to 0.55, directly correlating with the moisture content. It is essential to highlight that the water content present in the powders of roasted beans, used in the preparation of beverages, such as coffee, for example, is responsible for providing balanced acidity and a pleasant aroma to the product [22].

The powders from roasted açaí beans presented protein values ​​between 4.62 and 5.67 g/100 g. This protein fraction plays a fundamental role during the roasting process in the occurrence of biochemical reactions, such as the Maillard Reaction, for forming compounds associated with the aroma and flavor of the final product [6]. The lipid values ​​in roasted açaí grain powders ranged from 5.09 to 12.22 g/100 g, a notable variation that may significantly influence viscosity of the drink, as the natural oils present in the grains create a creamier infusion sensation in the mouth.

The powders' carbohydrate content represented the most significant fraction, with values ​​of 82.19–89.76 g/100 g. In addition to nutritional quality, carbohydrates' presence represents technological and sensory importance, as carbohydrates participate directly in the Maillard Reaction and Caramelization, which are responsible for the formation of flavor [23].

The ash content observed in the different samples ranged from 0.83 to 1.92 g/100 g. These values ​​are below those found for coffee, for example, for which ANVISA establishes a maximum limit of 5 g/100 g of sample for ground and packaged coffee. The different types of grain, initial chemical composition, or degree of roasting, can justify the variations in ash content found [24].

The samples were characterized as acidic due to their low pH value, which varied between 2.95 and 4.95. Acidity is also related to factors such as the place of origin of the fruits, forms of processing, types of drying, and climatic conditions. Sensorially, the acidity perceived in drinks such as coffee becomes a relevant sensory attribute for product choice [25].

Together, these findings highlight the current heterogeneity in the chemical composition of roasted açaí seed powders, evidencing the lack of standardization of the roasting processes, which can cause changes in the sensory and aromatic quality of the beverages obtained [20], as well as in the phenolic profile and functional properties.

### 5.2 Bioactive Evaluation Before and After Digestion (In Vitro) of Percolated Beverages Made With Roasted Açaí Beans

#### 5.2.1 Condensed Tannins and Total Flavonoids

**FIGURE 1:**
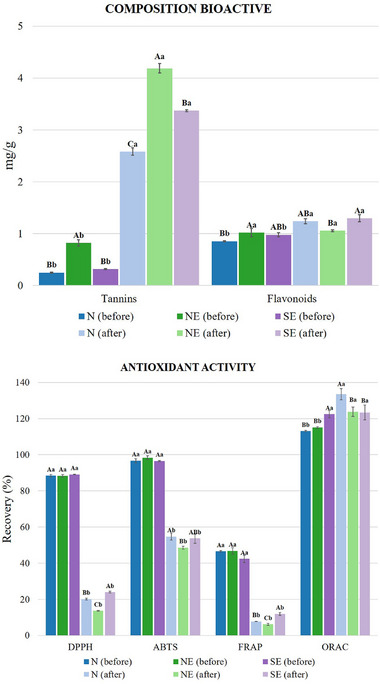
(A) Effect of simulated gastrointestinal digestion on the bioactive composition of roasted açaí grain percolate. The results expressed in this figure were statistically analyzed by performing mean ± standard deviation comparing the same sample, and between the regions before B and after A, the gastrointestinal digestion test. Samples before the digestive process can be identified with a darker color, and after with lighter colors. Different capital letters indicate differences between Brazilian regions for the same method, denote differences (*p* ≤ 0.05). Different lowercase letters show significant difference between the same sample before and after the digestive process based on *t*‐test. (B) Effect of simulated gastrointestinal digestion on the release of antioxidant compounds. The results shown in this figure were developed statistically and mean ± standard deviation comparing the same sample, and between the regions before B and after A, the gastrointestinal digestion test. Samples before the digestive process can be identified with darker coloring, and after in lighter nuclei. Different letters denote differences between Brazilian regions for the same method (*p* ≤ 0.05). Different lowercase letters show a significant difference between the same sample before and after the digestive process using the *t*‐test.

The tannin values found in the samples varied from 0.24 to 0.82 mg/g of powder, emphasizing the sample produced in Brazil's Northeast region (NE‐PE) (Figure 1A). Sensorially, tannins are characterized by their astringent flavor, which is predominant and striking in percolated drinks produced with roasted açaí grain powders. Furthermore, tannins also demonstrate anti‐inflammatory, anticancer, antimicrobial, and antioxidant action, helping to reduce and protect cells from harmful effects [26, 27].

Like tannins, flavonoids also help maintain and preserve the inflammatory response, and they can reduce the risk of cardiovascular diseases while having a therapeutic effect on hypertension [28]. In the analyzed samples of roasted açaí grains, flavonoid concentrations were determined between 0.85, 0.97, and 1.02 mg/g for the N (AP), SE (SP), and NE (PE) regions, respectively. Using açaí pulp and seeds as emerging sources of phenolic compounds, Rossetto et al. [29] found high concentrations of flavonoids, around 31% of the total phenolic compounds.

However, changes in the chemical profile and concentrations of compounds such as tannins and flavonoids may occur during gastrointestinal digestion. Due to this, it is essential to evaluate these compounds during digestion and bioaccessibility. As shown in Figure 1A, the values of condensed tannins after the in vitro digestion process were higher than those obtained before digestion in all samples evaluated, with emphasis on the sample produced in the North of the country (N‐AP) (5.72 mg/g of powder). This increase may be related to its high molecular weight and flexible tannin chains, enabling possible connections with other compounds to generate complexes [12].

Total flavonoids increased after digestion, with values that varied from 1.06 mg/g (sample produced in the Northeast—NE (PE) to 1.29 mg/g (sample created in the Southeast—SE (SP) The increase in the concentration of phenolic compounds, such as tannins and flavonoids, after the digestion process, may be related to the prolonged digestion time and the changes in the pH value that occur in this process. Degraded, there is a loss of interaction between carbohydrates and phenols, generating the release of phenols and causing a significant increase in these compounds at the end of digestion [30].

#### 5.2.2 Antioxidant Activity

The samples were also evaluated for their antioxidant effect against DPPH, ABTS, iron reduction (FRAP) radicals, and oxygen radical absorption capacity (ORAC) (Figure 1B). There were no significant differences in the samples before digestion regarding the action of the DPPH radical (∼88%) and ABTS (96% to 98%). Antioxidant activity of roasted açaí beverages was higher than coffee (DPPH 70%) and green tea (DPPH 82%) [31, 32]. Green tea inhibition ranged from 70% to 20%, varying by raw material, extraction, and storage [33]. Samples from North (N) and Northeast (NE) showed greater FRAP activity. Reducing power is linked to tannins, phenolic acids, and flavonoids [34].

ORAC ranged from 115% to 122%, exceeding Trolox standard. Evaluating three varieties of teas, Huang et al. [35] observed that samples of teas prepared with hot water had an absorption capacity between 38% and 105%.

After digestion, DPPH (13%–23%), ABTS (48%–54%), and FRAP (4%–46%) decreased. This reduction may be associated with the susceptibility to degradation or transformation of phenolic compounds due to adverse pH and enzyme conditions in digestion [36].

However, ORAC remained stable (123%–133%). This result suggests the presence of stable phenolics (e.g., chlorogenic acid, proanthocyanidins) in açaí seeds [5]. Similar results found in fermented tea leaves: DPPH 22%, ABTS 26% [37]. In another study, Donlao and Ogawa [38] reported 16%–25.7% reducing power in digested green tea. Due to the stability of the compounds under complex gastrointestinal conditions, their bioavailability has been more extensively evaluated.

Analyzing the effects of digestive enzymes and pH of green tea during in vitro digestion, researchers observed a reduction in the antioxidant activity of FRAP after the gastrointestinal tract due to enzymatic actions and changes in pH. Studies indicate that Compound changes in digestion relate to structure and phytochemical stability [39]. After digestion, DPPH, ABTS, and FRAP declined, but ORAC remained stable, indicating that beverages obtained with roasted açaí grains can have significant antioxidant effects after ingestion.

#### 5.2.3 Total Phenolic Content and Phenolic Profile

The fresh açaí seeds presented a total phenolic content of 20.04 mg GAE/g of powder before digestion, with a significant reduction (4.70 mg GAE/g of powder) after in vitro digestion. This substantial reduction can be attributed to the susceptibility of certain phenolics, such as flavanols and procyanidins, to degradation by digestive enzymes and alkaline conditions, as well as to their interaction with the dietary fiber and proteins naturally present in the fresh seeds [4, 5].

In contrast, roasted grain powders presented lower total phenolic values even before digestion, ranging from 1.68 to 2.60 mg GAE/g depending on the region of origin (North: 1.68–2.60 mg GAE/g; Northeast: 1.72 mg GAE/g; Southeast: 1.97 mg GAE/g). This is likely explained by the degradation of phenolic compounds by heat during the roasting process, leading to the formation of melanoidins that can incorporate phenolic acids into their structure, thereby increasing stability but reducing the quantification of free phenolics. Thus, these results indicate that both processing (roasting) and gastrointestinal tract conditions impact the availability and bioaccessibility of phenolic compounds, with raw beans showing greater overall retention of phenolic compounds after digestion compared to roasted samples [29, 30].

**FIGURE 2:**
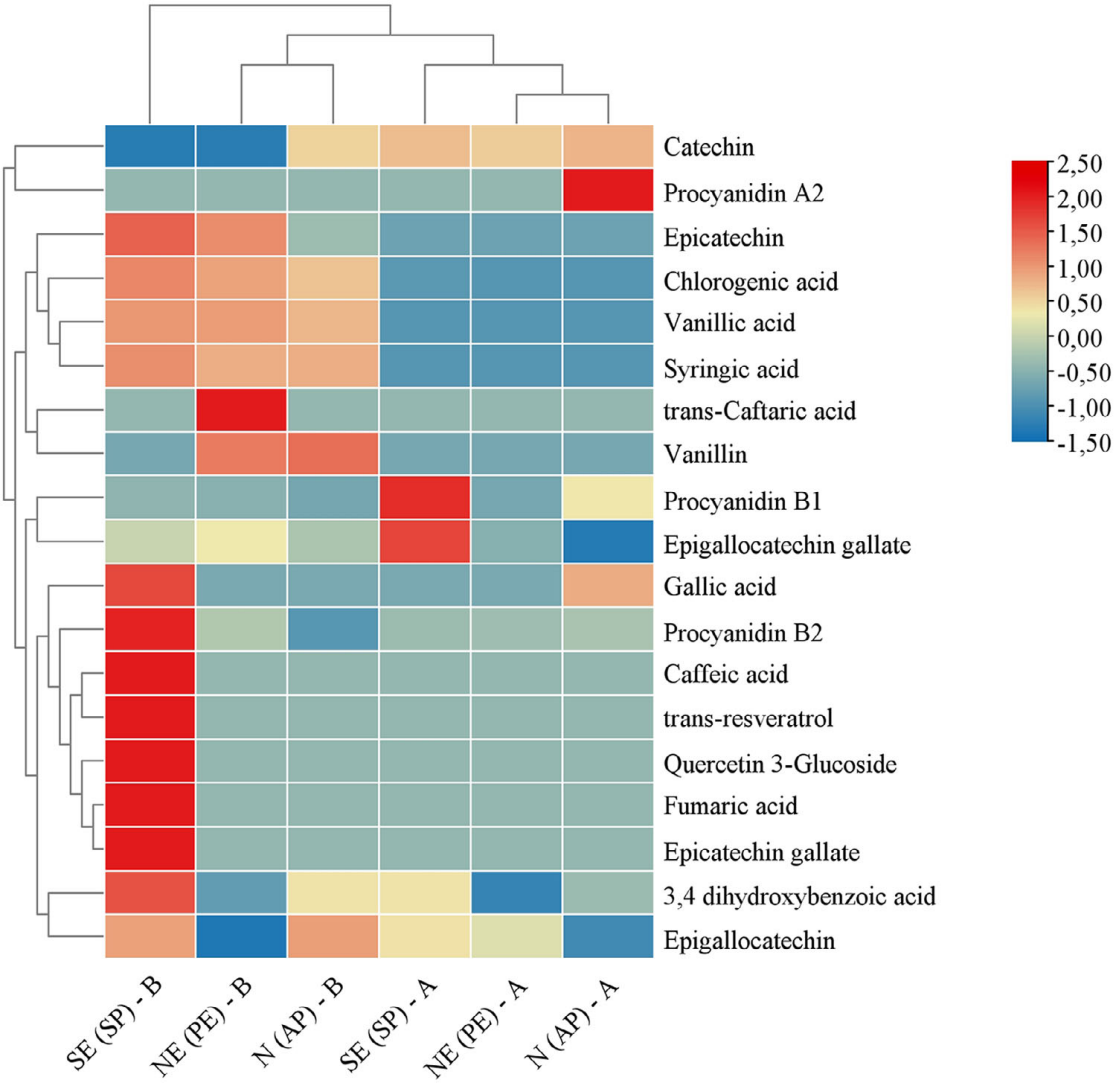
Heatmap and hierarchical cluster of the phenolic compound profile of roasted açaí grain percolate. The first three columns (SE‐SP‐B); (NE‐PE‐B); (N‐AP‐B) refer to the samples before digestion, and the three following (SE‐SP‐A); (NE‐PE‐A); (N‐AP‐A) after gastrointestinal simulation.

In addition, 19 phenolic compounds were identified and quantified before and after the simulated digestion of beverages made from roasted açaí beans: 7 phenolic acids, 6 flavonoids, 3 tannins, 2 simple phenols, and 1 fumaric acid. The hierarchical clustering analysis (HCA) of the phenolic compound profile can be seen in Figure 2.

Two significant clusters were formed, separating the samples before and after digestion, indicating changes in the phenolic compound profile during the digestive process. This suggests that phenolic compounds may transform the simulation of passage through the gastrointestinal tract due to alterations caused by digestive enzymes and chemical reactions, leading to the formation of bioactive metabolites or partial degradation. Additionally, biomolecules present in food can affect the availability of phenolics, such as complex carbohydrates that directly interact with phenolic compounds, interfering with their extraction and proper quantification [40].

When evaluating the samples before simulated digestion, it was observed that those produced in the North (N‐AP) and Northeast (NE‐PE) regions of Brazil exhibited a similar phenolic profile compared to the sample from the Southeast region (SE‐SP). The NE‐PE and N‐AP samples showed higher proportions of syringic, vanillic, chlorogenic acids, and epicatechin. In contrast, the SE‐SP sample was characterized by a predominant presence of epicatechin gallate, fumaric acid, caffeic acid, trans‐resveratrol, quercetin 3‐glucoside, procyanidin B2, gallic acid, and 3,4‐dihydroxybenzene acid.

The differences between the samples can be explained by environmental factors (soil and photoclimatic conditions) and processing methods. Brazil's northern and northeast regions have higher temperatures than the southeast, which is characterized by a colder climate, resulting in alterations in the chemical characteristics of the beans both before and after the roasting process. Moreover, the lack of standardization in açaí roasting, with variations in process time and temperature, directly contributes to the variability of phenolic profiles. The Maillard and caramelization reactions, intensified by roasting time and temperature, can modify the composition of phenolics in the prepared beverages and promote the formation of bioactive compounds such as melanoidins. The interaction of melanoidins with phenolic compounds increases their stability or promotes their conversion into more bioavailable forms [41].

High molecular weight melanoidins exhibit antioxidant, antimicrobial, and antihypertensive activity. These bioactivities may be enhanced due to the incorporation of chlorogenic acids into their structure during roasting. Along with temperature, time plays a crucial role in phenolic composition, either increasing or decreasing their concentration [42]. When evaluating the effects of roasting on the antioxidant activity of different coffees, Kautzmann et al. [43] observed that, depending on roasting conditions, the antioxidant potential is affected, indicating that the process influences the reduction of activity.

Analyzing the percolations made with roasted açaí beans from the N (AP), NE (PE), and SE (SP) regions after in vitro digestion, higher proportions of catechin, procyanidin A2 and B1, and epigallocatechin gallate were observed. The increased presence of these compounds after digestion is related to the degradation and biotransformation of previously formed compounds, such as procyanidin B1, composed of epicatechin‐(4β‐8)‐catechin, as well as procyanidin A2, formed by catechin and an epicatechin unit (2β→O→7, 4β→8). These flavonoids and gallic acid are predominant in fresh açaí beans [5], demonstrating that part of these compounds remains stable during processing and digestion.

All analyzed percolations showed a reduction in the content of vanillic, syringic, fumaric, chlorogenic, and caffeic acids, epicatechin, epicatechin gallate, trans‐caftaric acid, vanillin, trans‐resveratrol, and quercetin 3‐glucoside. The reduction in these compounds may be associated with enzymatic degradation or the alkaline pH of pancreatic juice, which promotes oxidation and conjugation reactions, reducing the concentration of phenolics in the bioaccessible fraction [44].

The ability of some compounds to remain intact or even increase after digestion depends on several factors related to chemical structure, resistance to digestive enzymes, and acidic environment. An example is catechins and gallocatechins. These flavonoids can influence protein utilization by forming complexes with proteins and iron, binding strongly with peptides, resisting digestion [45].

A study evaluating the metabolic benefits of green tea catechins at the intestinal level showed that this compound plays a crucial role in metabolic benefits by influencing processes, aiding in functional intestinal barrier maintenance, and reducing mucosal inflammation [46]. It acts directly at the intestinal level, improving function, strengthening cells, enhancing nutrient absorption, and preventing intestinal pathologies [26].

Overall, the phenolic compounds found in the percolations after digestion provide health benefits to the host when adequately consumed. Therefore, intestinal health is key in promoting well‐being and is influenced by external factors and dietary patterns.

#### 5.2.4 Principal Component Analysis

**FIGURE 3:**
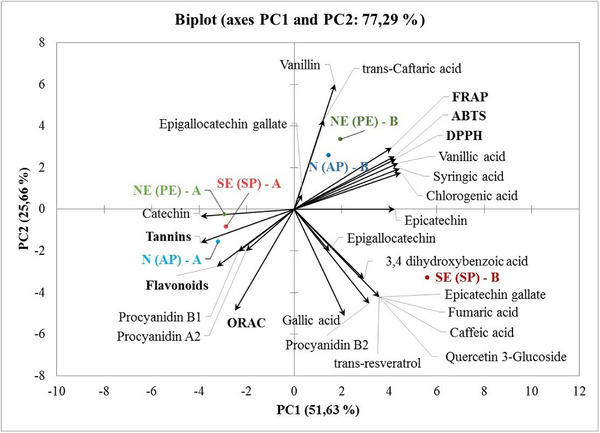
Analysis of the main components of the phenolic profile, bioactive composition, and antioxidant activity in hot roasted açaí grain drinks before and after simulated gastrointestinal digestion. The samples SE‐(SP)‐B; NE‐(PE)‐B; N‐(AP)‐B refer to the samples before digestion, and the samples SE‐(SP)‐A; NE‐(PE)‐A; N‐(AP)‐A correspond to the gastrointestinal simulation process.

The results of the bioactive evaluation regarding the antioxidant activity of the percolations of roasted açaí grains before and after digestion were analyzed using Principal Component Analysis (Figure 3). The first principal component (PC1) explained 52.63% of the total variation between the samples, and the second principal component (PC2) explained 25.66% of the total variability. PC1 × PC2 explained 77.29% of the variability between the components evaluated.

A separation was observed between the samples before and after the digestive process. In the positive quadrant of PC1 and PC2, there was a grouping between the samples produced in the North and Northeast, close to the region of the graph where the antioxidant activity vectors (FRAP, ABTS, and DPPH) are found. In the same quadrant are the chlorogenic acids, vinylic acid, and syringic acid, which are associated as phenolic markers for the potential antioxidant activity of these samples.

Because they are susceptible throughout the gastrointestinal digestion process and consequently modify the quantity and profile of phenolic compounds, it was possible to observe that simulated digestion positively affected the amount of tannins and flavonoids (catechin and procyanidins), which in turn are associated with increased antioxidant activity by the ORAC method. These compounds are linked to benefits such as the prevention of chemoprotective, thermogenic, anti‐inflammatory, and anticarcinogenic diseases [47].

## 6 Cytotoxic/Cytoprotective Assays (Caco‐2 Cell Culture)

### 6.1 Evaluation of Cell Viability With MTT Reduction Assay

**FIGURE 4:**
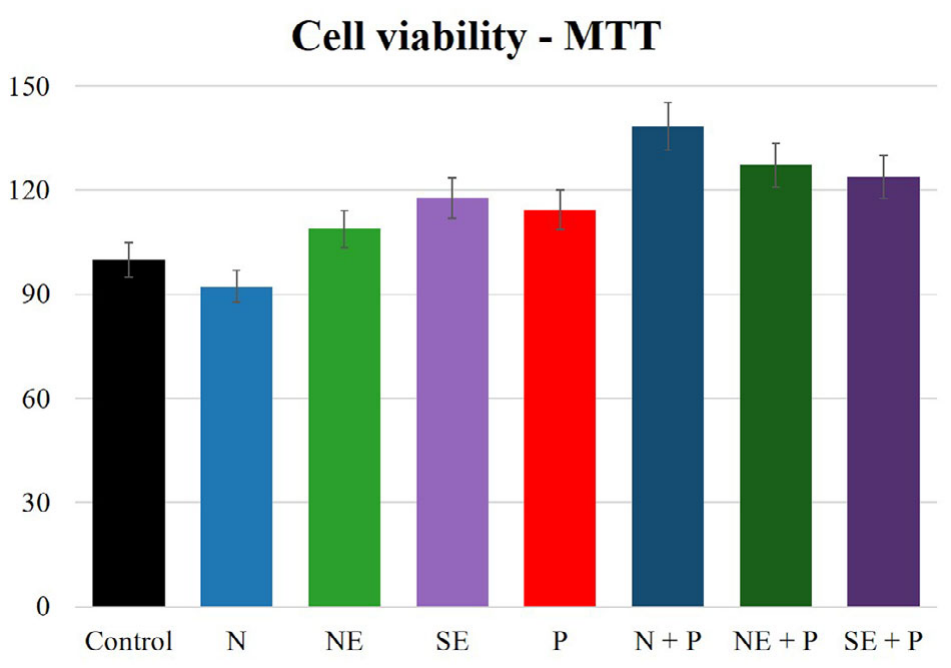
Cytotoxicity test using roasted açaí grain percolate. The results did not indicate statistically significant differences (*p* < 0.05). Equal letters indicate that there was no significant difference (*p* < 0.05) in the percolate (N‐AP‐B; NE‐PE‐B; SE‐SP‐B) and after being exposed to H_2_O_2_ (N‐AP‐A‐H_2_O_2_; NE‐PE‐A‐H_2_O_2_; SE‐SP‐A‐H_2_O_2_).

The toxicity of roasted açaí beans was assessed by analyzing the effects of percolation after the digestive process on the viability of Caco‐2 cells using the MTT assay. All the values obtained were higher than 90%, suggesting that the tested samples have low cytotoxicity. The result (Figure 4) showed that the sewage was not toxic to Caco‐2 cells at a concentration of 0.500 µg/mL or when incubated with H_2_O_2_ and did not affect their viability. This shows that the compounds in the sludge do not cause damage to the functions of Caco‐2 cells.

Performing an MTT assay on coffee with Caco‐2 cells, Bravo et al. [48] reported results from cells with coffee infusions that showed that at the highest dose, there were no cytotoxic effects (500 µg/mL); on the other hand, when exposed to H_2_O_2_, there was a significant reduction in cell viability. In another study using extracts of Robusta and Arabica coffees, Grzelczyk et al. [7] found that preparations obtained with Arabica coffee were less cytotoxic than Robusta coffee within the concentration range of 0–500 µg/mL. The cytotoxic potential is correlated with the degree of roasting due to the high temperatures and exposure time, leading to lower concentrations of phenolic compounds and antioxidant activity.

### 6.2 Evaluation of ROS Production With DCF‐DA Assay

**FIGURE 5:**
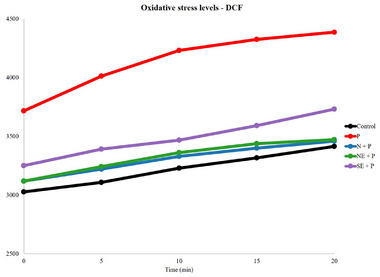
Cytoprotection assay using roasted açaí grain percolate after the digestive process with stimulation in Caco‐2 cells.The acronym composed of the suffix H_2_O_2_ (N‐AP‐A‐H_2_O_2_; NE‐PE‐A‐H_2_O_2_; SE‐SP‐A‐H_2_O_2_) indicates exposure to hydrogen peroxide.

The cytoprotective effect in the DCF‐DA assay (Figure 5) demonstrates that treating Caco‐2 cells with percolations of roasted açaí grains with oxidative stimulation showed that the samples prevented oxidation. In general, protection against reactive oxygen species is observed, demonstrating that the treatments performed with samples from different regions of Brazil have protective compounds (antioxidants), especially in the southeast region (SE), capable of acting as hydrogen donors and consequently protecting the cells from oxidative damage.

Corroborating the results of the present study, Grzelczyk et al. [7] showed that the results from green coffee preparations had a greater effect, reducing the level of ROS in Arabica coffee and resulting in greater efficiency compared to Robusta coffee.

Silva et al. [46] evaluated coffee extracts' protective and antioxidant effects. After inducing oxidative stress with H_2_O_2_, they described that coffee extracts could reduce ROS production at the highest concentrations (333–1000 µg/mL).

The amount of phytocompounds in roasted açaí beans capable of reducing oxidative stress makes the product a new beverage of industrial and commercial interest. These results may be related to the antioxidant properties present in the grains, such as phenolic compounds, such as gallic acid and melanoidins, resulting from the Maillard reaction and caramelization formed during the product roasting process [5].

## 7 Concluding Remarks

Beverages made with roasted açaí berries, produced and sold in Brazil, are emerging as an upcycling product. They add value to the byproducts of the açaí production chain and contribute to more sustainable food systems. This beverage is promising, as it is a source of phenolic compounds with the potential to promote benefits to human health and attract the food industry.

The beverages' chemical composition was influenced by the geographic region and the lack of standardization of the roasting process, which directly impacted the content of phenolic compounds and consequent bioactivity. After the in vitro digestion process, they presented a change in the phenolic profile with effective antioxidant potential, acting against reactive oxygen species (ROS). In addition, all samples showed low cytotoxicity in Caco‐2 cells and cytoprotective effect, reinforcing their functional potential.

This study pioneered the mapping of the chemical and bioactive properties of roasted açaí bean powders available on the Brazilian market. Future studies should explore in vivo biological models to optimize the development of functional products based on roasted açaí beans and optimize roasting parameters to improve the quality and development of this beverage.

## Supporting information




**Supporting File 1**: mnfr70270‐sup‐0001‐TableS1.Docx

**Supporting File 2**: mnfr70270‐sup‐0002‐TableS2.Docx

## Data Availability

The authors have nothing to report.
