# Supplementary material for: Upcycled Beverage From Roasted Açaí (Euterpe oleracea) Seeds: Antioxidant Capacity and Cytoprotection Through Gastrointestinal Simulation
Source: Mol Nutr Food Res. 2025 Sep 16;69(23):e70270. doi: 10.1002/mnfr.70270 (PMC12666764; doi:10.1002/mnfr.70270)
Supplement: Supplementary file 1 — Supporting File 1: mnfr70270‐sup‐0001‐TableS1.Docx [file MNFR-69-e70270-s002.docx]

**Supplementary**

Table S1: Total phenolic compounds (mg GAE/g).

| SAMPLES | | | | | |  |
| --- | --- | --- | --- | --- | --- | --- |
| NE(PE) | N(AP1) | N(AP2) | N(AP3) | N(PA) | SE(SP) | |
| 1,72±0,05 ^a^ | 2,45 ±0,07 ^a^ | 1,84±0,10 ^b^ | 2,60±0,05 ^a^ | 1,68±0,05^b^ | 1,97±0,002 ^a^ | |

NE(PE): Northeast (Pernambuco). N(AP1): North (Amapá 1). N(AP2): North (Amapá 2). N(AP3): North (Amapá 3). N(PA): North (Pará). SE(SP): Southeast (São Paulo). The results were expressed as mean ± standard deviation. Different letters denote differences (p ≤ 0.05), based on Tukey's test.
