# Supplementary material for: Upcycled Beverage From Roasted Açaí (Euterpe oleracea) Seeds: Antioxidant Capacity and Cytoprotection Through Gastrointestinal Simulation
Source: Mol Nutr Food Res. 2025 Sep 16;69(23):e70270. doi: 10.1002/mnfr.70270 (PMC12666764; doi:10.1002/mnfr.70270)
Supplement: Supplementary file 2 — Supporting File 2: mnfr70270‐sup‐0002‐TableS2.Docx [file MNFR-69-e70270-s001.docx]

**Table S2.** Method validation parameters for phenolic determinations by RP-HPLC/DAD.

| Phenolic compounds | Retention time | λ (nm) | Range mg/L (n=5) | Linearity R^2^ | Precision CV% | Recovery % | LOD mg/L | LOQ mg/L |
| --- | --- | --- | --- | --- | --- | --- | --- | --- |
| Fumaric acid | 1.43 | 220 | 0.5-10 | 0.999 | 1.48 | 87.1 | 0.23 | 0.52 |
| Gallic acid | 2.10 | 280 | 0.5-10 | 0.999 | 1.44 | 87.6 | 0.27 | 0.53 |
| 3,4-dihydroxybenzoic acid | 3.36 | 280 | 0.5-10 | 0.999 | 1.23 | 96.7 | 0.18 | 0.27 |
| trans-Caftaric acid | 4.88 | 320 | 0.5-10 | 0.999 | 6.28 | 88.8 | 0.16 | 0.45 |
| Procyanidin B1 | 4.89 | 220 | 0.5-15 | 0.998 | 1.13 | 103.8 | 0.11 | 0.23 |
| Epigallocatechin gallate | 5.22 | 220 | 0.5-5.0 | 0.999 | 6.5 | 100.7 | 0.30 | 0.45 |
| Catechin | 5.50 | 220 | 0.5-10 | 0.999 | 2.73 | 89.6 | 0.13 | 0.41 |
| 4-hydroxybenzoic acid | 6.02 | 280 | 0.5-10 | 0.999 | 6.35 | 87.5 | 0.10 | 0.35 |
| Vanillic acid | 6.32 | 280 | 0.5-10 | 0.999 | 0.47 | 93.4 | 0.15 | 0.42 |
| Caffeic acid | 6.51 | 320 | 0.5-10 | 0.999 | 2.82 | 93.2 | 0.22 | 0.56 |
| Cholorogenic acid | 6.64 | 320 | 0.5-10 | 0.998 | 2.33 | 98.1 | 0.32 | 0.60 |
| Procyanidin B2 | 6.82 | 220 | 0.5-15 | 0.999 | 4.25 | 102.2 | 0.13 | 0.35 |
| Vanillin | 7.49 | 320 | 0.5-10 | 0.999 | 0.45 | 89.9 | 0.15 | 0.34 |
| Syrungic acid | 7.88 | 280 | 0.5-10 | 0.999 | 3.32 | 105.6 | 0.41 | 0.86 |
| Cyanidin 3,5-diglucoside | 8.29 | 520 | 0.5-15 | 0.999 | 4.78 | 92.1 | 0.65 | 0.95 |
| Epicatechin | 8.40 | 220 | 0.5-15 | 0.999 | 3.01 | 85.9 | 0.12 | 0.38 |
| p-Coumaric acid | 9.00 | 320 | 0.5-10 | 0.999 | 2.23 | 96.3 | 0.21 | 0.49 |
| Pelargonidin 3,5-diglucoside | 9.44 | 520 | 0.5-10 | 0.999 | 6.42 | 90.5 | 0.55 | 0.86 |
| Delphinidin 3-glucoside | 9.64 | 520 | 0.5-10 | 0.999 | 2.49 | 82.2 | 0.45 | 0.73 |
| orto-Vanillin | 10.09 | 360 | 0.5-10 | 0.999 | 2.92 | 89.9 | 0.58 | 1.12 |
| Ferulic acid | 10.99 | 320 | 0.5-10 | 0.999 | 0.41 | 98.4 | 0.12 | 0.35 |
| Cyanidin 3-glucoside | 11.00 | 520 | 0.5-15 | 0.999 | 6.13 | 86.1 | 0.48 | 0.54 |
| Malvidin 3,5-diglucoside | 11.21 | 520 | 0.5-10 | 0.999 | 5.64 | 93.7 | 0.58 | 0.75 |
| Epicatechin gallate | 11.32 | 220 | 0.5-5.0 | 0.998 | 4.47 | 103.5 | 0.29 | 0.55 |
| Procyanidin A2 | 12.35 | 220 | 0.5-15 | 0.999 | 5.97 | 84.9 | 0.23 | 0.45 |
| Pelargonidin 3-glucoside | 12.37 | 520 | 0.5-15 | 0.999 | 2.84 | 87.4 | 0.52 | 0.92 |
| Myricetin | 13.44 | 360 | 0.5-10 | 0.999 | 4.93 | 84.2 | 0.15 | 0.48 |
| Peonidin 3-glucoside | 13.73 | 520 | 0.5-10 | 0.999 | 5.21 | 92.4 | 0.44 | 0.84 |
| Malvidin 3-glucoside | 14.80 | 520 | 0.5-10 | 0.999 | 6.08 | 89.4 | 0.33 | 0.72 |
| trans-Resveratrol | 15.78 | 320 | 0.5-10 | 0.999 | 3.13 | 102.9 | 0.08 | 0.12 |
| Quercetin 3-glucoside | 16.01 | 360 | 0.5-10 | 0.999 | 5.22 | 100.8 | 0.12 | 0.35 |
| Rutin | 16.17 | 360 | 0.5-10 | 0.999 | 4.84 | 98.5 | 0.13 | 0.34 |
| Naringin | 16.43 | 280 | 0.5-10 | 0.999 | 6.82 | 97.7 | 0.15 | 0.52 |
| Hesperidin | 17.03 | 280 | 0.5-10 | 0.999 | 3.98 | 91.5 | 0.15 | 0.49 |
| trans-Cinnamic acid | 17.50 | 280 | 0.5-10 | 0.999 | 1.28 | 102.8 | 0.21 | 0.32 |
| Kaempferol 3-glucoside | 17.52 | 360 | 0.5-10 | 0.999 | 4.86 | 95.1 | 0.15 | 0.32 |
| cis-Resveratrol | 17.71 | 280 | 0.5-15 | 0.999 | 6.41 | 94.4 | 0.15 | 0.28 |
| Isorhamnetin | 17.91 | 360 | 0.5-5.0 | 0.999 | 3.68 | 91.8 | 0.18 | 0.35 |
| Quercetin Hydrate | 18.90 | 360 | 0.5-10 | 0.999 | 6.81 | 81.5 | 0.22 | 0.46 |
| Naringenin | 18.92 | 280 | 0.5-10 | 0.999 | 6.19 | 93.4 | 0.31 | 0.88 |
| Hesperitin | 19.75 | 280 | 0.5-10 | 0.999 | 6.15 | 101.5 | 0.26 | 0.82 |
